# Analysis of cancer-associated glycosyltransferases reveals novel targets of non-small cell lung cancer pathogenesis

**DOI:** 10.3389/fonc.2025.1601368

**Published:** 2025-07-11

**Authors:** Chang Liu, Thomas van Ee, Jurriaan Janssen, Ernesto Rodríguez, Yongsoo Kim, Teodora Radonic, Victor W. van Beusechem, Marieke F. Fransen, Idris Bahce, Yvette van Kooyk

**Affiliations:** ^1^ Pulmonary Medicine, Amsterdam UMC location Vrije Universiteit Amsterdam, Amsterdam, Netherlands; ^2^ Molecular Cell Biology and Immunology, Amsterdam UMC location Vrije Universiteit Amsterdam, Amsterdam, Netherlands; ^3^ Amsterdam Institute for Infection and Immunity, Amsterdam, Netherlands; ^4^ Cancer Center Amsterdam, Amsterdam, Netherlands; ^5^ Pathology, Amsterdam UMC location Vrije Universiteit Amsterdam, Amsterdam, Netherlands; ^6^ Medical Oncology, Amsterdam UMC location Vrije Universiteit Amsterdam, Amsterdam, Netherlands

**Keywords:** glycosylation, mucins, galectins, α2,6-GalNAc-sialylation, non-small cell lung cancer

## Abstract

**Background:**

Aberrant glycosylation is associated with cancer progression and patient survival, of which the driving genes could act as biomarkers. Our objective was to characterize the expression of glycosylation-related genes to elucidate the heterogeneity between lung adenocarcinoma (LUAD) and lung squamous cell carcinoma (LUSC), and their prospective diagnostic utility.

**Methods:**

mRNA expression data for all glyco-relevant genes was collected from 553 LUSC and 576 LUAD patients from the TCGA dataset. Differential gene expression analysis and UMAP dimension reduction analysis were used to compare mRNA expression in LUAD and LUSC. Selected genes were further confirmed through immunohistochemistry of tissue biopsies. Public single-cell RNA sequencing (scRNA-seq) data from 72 LUSC and 163 LUAD patients was retrieved to study cell type-specific expression. Galectin-7 was measured in patients’ plasma by ELISA. Univariate Cox proportional regression model was used for prognostic marker detection.

**Results:**

Our analysis revealed genes differentially expressed respectively in LUSC and LUAD compared to normal lung samples. We focused on genes exhibiting high expression in LUSC (*LGALS7*, *LGALS7B*, and *ST6GALNAC2*) and in LUAD (*LGALS4*, *MUC21*, and *ST6GALNAC1*). Key glyco-related signatures were mostly observed in the malignant cell compartment. Galectin-7 concentration in plasma was upregulated in LUSC patients, but not LUAD patients. 67 genes in LUAD and 23 genes in LUSC were strongly linked to patient survival.

**Conclusion:**

We identified several glyco-associated biomarkers in NSCLC, including Galectin-4, Galectin-7, MUC21, ST6GALNAC1, and ST6GALNAC2. Galectin-7 is a promising clinical biomarker for detection in plasma.

## Introduction

Lung cancer is one of the most common types of cancer and has the highest cancer-related mortality rate worldwide (1). Identifying tumor characteristics that are associated with poor prognosis may open possibilities for clinicians to tailor their treatment strategies. One such interesting cancer feature is protein glycosylation, which is the most frequent post-translational modification of cell surface proteins. Typically, cancer cells exhibit aberrant glycosylation, with certain glycan structures being associated with tumor invasiveness (2). For instance, abnormal O-glycosylation on the tumor cell surface is associated with poor prognosis and metastatic potential in lung cancer patients (3).

In this context, some families of glycosylation-associated genes–such as mucins, galectins, and sialyltransferases–have been shown to play a pivotal role in promoting tumor growth (4, 5). Mucins constitute a family of high-molecular-weight glycoproteins, playing a key role in the initiation and progression of various malignancies (4). Galectins are a group of carbohydrate−binding proteins, which are involved in lung cancer tumor growth. Galectins carry out their biological functions primarily through interactions with specific glycoconjugates (6). Aberrant sialylation promotes tumor progression through various mechanisms, such as stimulating tumor invasion and migration, as well as enhancing immune evasion (7). Human sialyltransferases (STs) are a family of glycosyltransferases that are responsible for sialic acid transfer from a nucleotide sugar donor (CMP-Neu5Ac) to the terminus of glycoproteins and glycolipids (8). According to the carbohydrate linkage between the sialic acid and the underlying glycan, STs can be classified into 4 families: the ST3Gal (α2,3-ST), ST6Gal (α2,6-ST), ST6GalNAc(α2,6-ST), and ST8Sia (α2,8-ST) families. Additionally, a series of genes (*GNE*, *NANS*, *NANP*, *CMAS*, and *SLC35A1*) generate donor synthesis enzymes involved in the biosynthesis and transport of CMP-Neu5Ac to the Golgi Apparatus.

In this study, our primary objective is to elucidate the profile of all glycosylation-associated genes in lung cancer for clinical application potential (9). We used transcriptomic analysis to identify genes differentially expressed in lung squamous cell carcinoma (LUSC), lung adenocarcinoma (LUAD) and normal lung, some of which were confirmed using immunohistochemistry (IHC). Furthermore, we evaluated several key glyco-related genes in various cell types using single-cell RNA sequencing (scRNA-seq) data, therefore clarifying their distributional heterogeneity in LUAD and LUSC. Moreover, we aimed to identify genes correlated with patient survival.

## Methods

### Transcriptomic analysis of glycosylation related genes in TCGA dataset

mRNA sequencing data from TCGA dataset was downloaded from the Genome Data General Database (GDC) data portal, which contains 553 patients with lung squamous cell carcinoma (LUSC) and 576 patients with lung adenocarcinoma (LUAD). Adjacent normal tissue samples were collected from patients with LUAD (n = 58) and LUSC (n = 51), and subsequently combined for downstream analyses. Clinical data were downloaded from the same source and matched to the processed lung TCGA data. Highly variable genes were selected based on the tool (http://pklab.med.harvard.edu/scw2014/subpop_tutorial.html). Upon doing calculations for estimates of variance and coefficient of variation of the bulk data, a total of 15252 genes were ranked based on the significance of deviation from the fit. The Wilcoxon test was used to identify differentially expressed genes (DEGs) in LUAD/LUSC compared to combined adjacent normal samples of LUAD and LUSC. DEGs were selected based on absolute binary logarithms of fold changes (Log_2_FC) >0.8 and false discovery rate (FDR) < 10^-5^. A previously published list of all glycosylation genes was used (10). Uniform Manifold Approximation and Projection (UMAP) was used for dimension reduction.

### Single-cell RNA sequencing analysis

The integrated scRNA-seq atlas from Salcher et al. (11) was downloaded, which consists of 1,283,972 cells from 318 patients. Cells which were annotated as originating from primary tumor sites and either LUAD or LUSC were selected, resulting in a dataset of 345,260 cells from 163 LUAD patients and 128,423 cells from 72 LUSC patients. After selecting cells, the UMAP space was recomputed using the *reprocess_adata_subset_scANVI* function (https://github.com/icbi-lab/luca) with default settings for visualization. Coarse cell type annotations with 12 cell types were adopted to study cell type specific-gene expression. For UMAP visualizations, gene expression counts were log-transformed and library size was corrected to 10,000 counts per cell. The Wilcoxon test was used to identify DEGs between LUAD and LUSC epithelial cells. To increase sensitivity of DEG identification in scRNA-seq data (12), pseudobulk mixtures were generated by aggregating counts of epithelial cells from each patient. Subsequently, pseudobulk mixtures were library size-corrected to 10,000 counts per mixture. DEGs between LUSC and LUAD pseudobulk mixtures were identified based on absolute binary logarithms of fold changes (Log_2_FC) >0.8 and false discovery rate (FDR) < 10^-2^.

### IHC staining

Immunohistochemical (IHC) staining of tissues was performed on FFPE sections (4 µm). Assisted by a pathologist, FFPE-tissue samples were selected from stage 3 LUAD (n=5) or LUSC (n=5) patients that underwent surgery prior to chemo, radio- and/or immunotherapy at the Vrije Universiteit medical center in Amsterdam. Paired adjacent non-malignant tissues were incorporated as reference controls. Ethical approval was not mandatory for this study due to the usage of leftover patient material, as stated in dossier number 2021.0063-VIP which was issued by the aforementioned medical center. Resected material was processed using conventional FFPE-tissue preservation techniques within clinical pathology labs.

Tissue slides were deparaffinized using xylene and rehydrated using ethanol, washed using demineralized water prior to heat induced antigen retrieval (DAKO Agilent, K800521–2 or K800421-2). Endogenous peroxidase activity was blocked by peroxidase-blocking solution (DAKO Agilent, S202386-2) for 10min and aspecific binding to tissue and Fc-receptors was blocked using protein block (Immunologic, VWRKBD09-999). Primary antibody (**Supplementary Table S1**) was dissolved in aforementioned protein block solution and incubated for 1 hour at room temperature, except for ST6GALNAC6 which was incubated overnight (20 hours) at 4°C, further details are listed in **Supplementary Table S1**. After incubation with the primary antibody, slides were incubated with BrightVision Poly-HRP-Anti Mouse/Rabbit IgG Biotin-free (Immunologic, VWRKDPVO55HRP) at room temperature for 30 mins. Antibody targets were visualized using DAB (3,3’-diaminobenzidine) for 10 minutes and slides were counterstained with hematoxylin (Sigma-Aldrich, 51275) and embedded with entellan (Sigma-Aldrich, 1079610500).

### Imaging

Stained slides were imaged at a 40x magnification on the Vectra Polaris Automated Quantitative Pathology Imaging System (Akoya Biosciences, software version 1.0.13). To remove anthracosis aspecific signal, acquired images were processed using inForm^®^ Tissue Analysis (Akoya Biosciences, software version 2.6.0) with a brightfield spectral library.

### Survival analysis

For the systematic analysis of survival, patients were stratified in high (top 25%) and low (bottom 25%) according to the expression of each glycosylation-related gene. The hazard ratio (HR), calculated in a univariate Cox proportional regression model analysis, was used to select genes that affect the prognosis, which were plotted in Kaplan Meier curves and significance studied using log-rank test.

### ELISA staining

Plasma from patients with lung adenocarcinoma (n=20) and Squamous cell carcinoma (n=16) was obtained from the Liquid Biopsy Center of the Amsterdam UMC. The studies involving human participants were reviewed and approved by the Medical Ethical Committee, Amsterdam UMC. Written consent was obtained from all the donors. A Galectin-7 ELISA kit (R&D Systems, DY1339) was used to measure its concentration in the plasma of patients. A standard curve based on recombinant Galectin-7 was used for quantification, with a blank control included to subtract background signal.

### Statistical analysis

R v4.3.0 software (https://mirror.lyrahosting.com/CRAN/) as used for statistical analysis and figure drawing. Significance was called when the adjusted *p*-value < 0.05. Key clinical characteristics of the patient cohort (TCGA datasets, single-cell datasets, IHC staining tissues, and ELISA sample sets) are summarized in **Supplementary Table S5**.

## Results

### Landscape of glyco-associated genes in NSCLC

**Figure 1:**
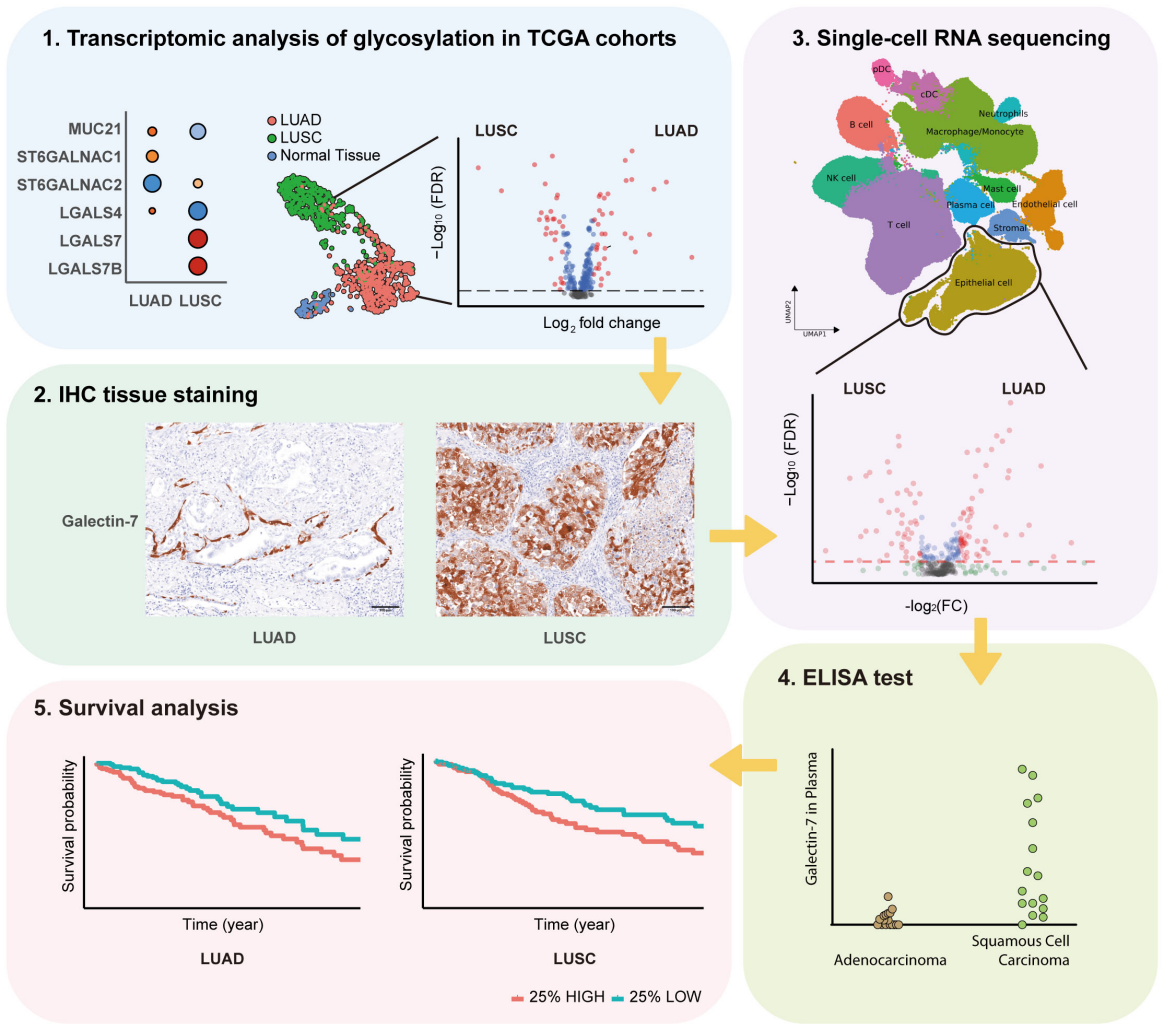
Flow chart of this study.

**Figure 2:**
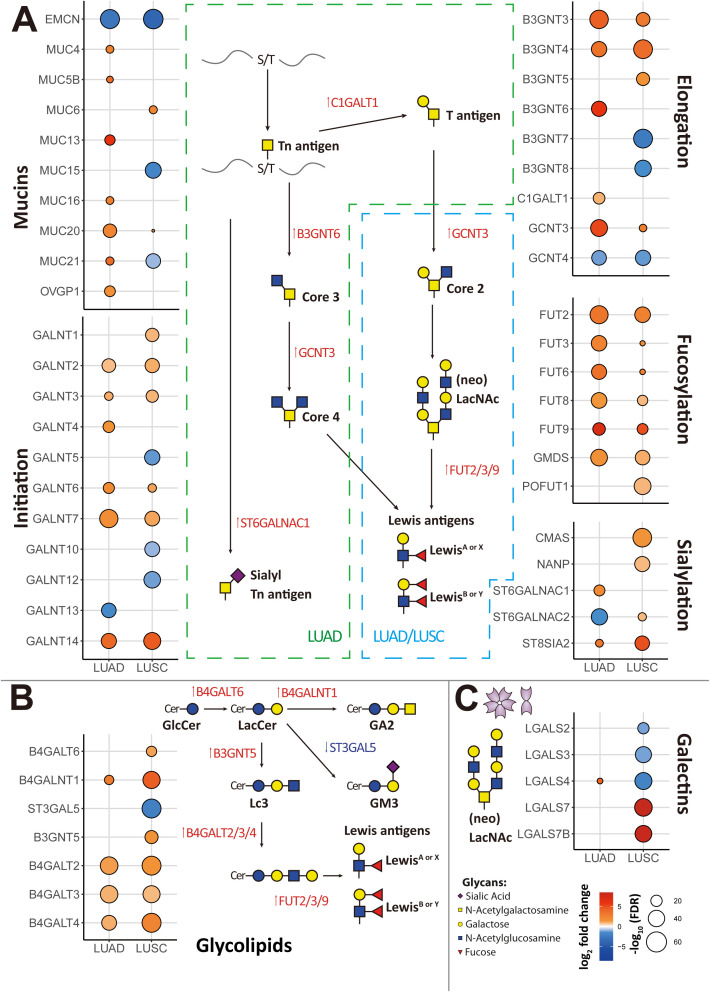
Signature of all differentially expressed genes (DEGs) associated with glycosylation in lung cancer from the RNA-Seq TCGA data. **(A)** DEGs associated with mucins or mucin-like proteins, initiation of GalNAc-type O-glycosylation, elongation, fucosylation, and sialylation were identified in adenocarcinoma (LUAD) and squamous cell carcinoma (LUSC) compared to adjacent normal samples. **(B)** DEGs associated with glycosphingolipid biosynthesis were identified in LUAD and LUSC compared to adjacent normal samples. **(C)** DEGs of galectins were identified in LUAD and LUSC compared to adjacent normal samples. Genes with an absolute value of log_2_ fold change > 0.8 were screened out. Highly expressed genes were plotted in red and low expressed genes were in blue based on log_2_ fold change value. False Discovery Rate (FDR) < 10–^5^ was considered statistically significant.

The study’s flow chart is shown in **Figure 1**. In order to investigate the expression of glycosylation-related genes, we started by analyzing differential gene expression between the different lung cancer subtypes and adjacent normal tissue (**Supplementary Table S2**, **Supplementary Table S3**). To facilitate the interpretation of the results, we grouped the results based on their involvement in different glycosylation pathways, including mucins, GalNAc-initiation, elongation, fucosylation, and sialylation (**Figure 2A**).

We observed that LUAD was particularly enriched in genes encoding for mucins or mucin-like proteins, in particular *MUC4*, *MUC5B*, *MUC13*, *MUC16*, *MUC20*, *MUC21*, and *OVGP1* (Oviductal glycoprotein 1) that were highly expressed in LUAD compared to normal lung, while *EMCN* was downregulated in LUAD. In LUSC, only *MUC6* and *MUC20* were upregulated, while *EMCN*, *MUC15*, and *MUC21* were downregulated.

Regarding initiation of GalNAc-type O-glycosylation, *GALNT2*, *GALNT3*, *GALNT6*, *GALNT7*, and *GALNT14* were highly expressed in both cancer types, LUAD and LUSC. In addition, *GALNT4* was also highly expressed in LUAD and *GALNT1* was also highly expressed in LUSC.

As for elongation, *B3GNT3*, *B3GNT4*, and *GCNT3* were highly expressed in both cancer types (LUAD and LUSC). *B3GNT6* and *C1GALT1* were highly expressed, and *GCNT4* was downregulated in LUAD. *B3GNT5* was upregulated, while *B3GNT7*, *B3GNT8*, and *GCNT4* were downregulated in LUSC. These results suggest that LUAD and LUSC both present an increase of O-glycans. We found several glycosylation elongation genes essential in the progress of synthesizing **core 3** (GlcNAcβ1–3GalNAc) and **core 4** (GlcNAcβ1–6[GlcNAcβ1–3]GalNAc) structures highly expressed in LUAD, including *C1GALT1*, *B3GNT6*, and *GCNT3*.

After aggregating all sialylation associated genes, we found *CMAS* and *NANP* were upregulated in LUSC. In the context of α2,6-GalNAc-sialylation, *ST6GALNAC1* exhibited upregulation in LUAD, while *ST6GALNAC2* showed downregulation in LUAD but upregulation in LUSC. Additionally, for α2,8-sialyltransferases, we found *ST8SIA2* was elevated in these two primary subtypes of NSCLC, with much higher expression in LUSC. However, we did not identify any upregulated genes compared to adjacent normal tissue among other sialyltransferases (**Supplementary Table S2**, **Supplementary Table S3**).

Genes associated with fucosylation, *FUT2*, *FUT3*, *FUT6*, *FUT8*, *FUT9*, and *GMDS* were upregulated in both LUAD and LUSC, although more significantly in the former. Especially in LUSC, we found an upregulation of *POFUT1*, encoding for an enzyme involved with O-fucosylation. In both LUAD and LUSC, the expression of fucosyltransferase gene *FUT2* was increased, facilitating the attachment of α1,2-fucosylation to Gal-residues. Moreover, within the same fucosyltransferase family, *FUT3*, *FUT6*, and *FUT9*—which facilitate α1,3- and α1,4-fucosylation of GlcNAc—along with *FUT8*, responsible for α1,6 core fucosylation of N-glycans, also exhibited upregulation. Interesting for the **Galectin family**, *LGALS4* was highly expressed in LUAD. Oppositely *LGALS7* and *LGALS7B* were upregulated, and *LGALS2*, *LGALS3*, and *LGALS4* were downregulated in LUSC (**Figure 2C**).

For glycolipid biosynthesis (**Figure 2B**), in both LUAD and LUSC, upregulation of *B4GALT2*, *B4GALT3*, and *B4GALT4*, pivotal genes integral to the synthesis of glycoproteins and glycolipids, was observed. These genes play a crucial role in facilitating the transfer of galactose during the growth of carbohydrate chains. GalNAc transferase *B4GALNT1* was upregulated both in LUAD and LUSC during the synthesis of ganglioside sugar structure GA2. Especially in LUSC, *B4GALT6* and *B3GNT5* were upregulated, which involved in the transfer of galactose and GlcNAc during the synthesis of glycoproteins and glycolipids. Given that there is a general increase of fucosylation in tumor, these glycolipids may also be fucosylated to generate Lewis antigens. *ST3GAL5*, which participates in the transfer of sialic acid (Neu5Ac) to galactose-containing substrates and catalyzes the formation of ganglioside GM3 using lactosylceramide (LacCer) as the substrate, was downregulated in LUSC.

### DEGs identification in LUAD and LUSC

**Figure 3:**
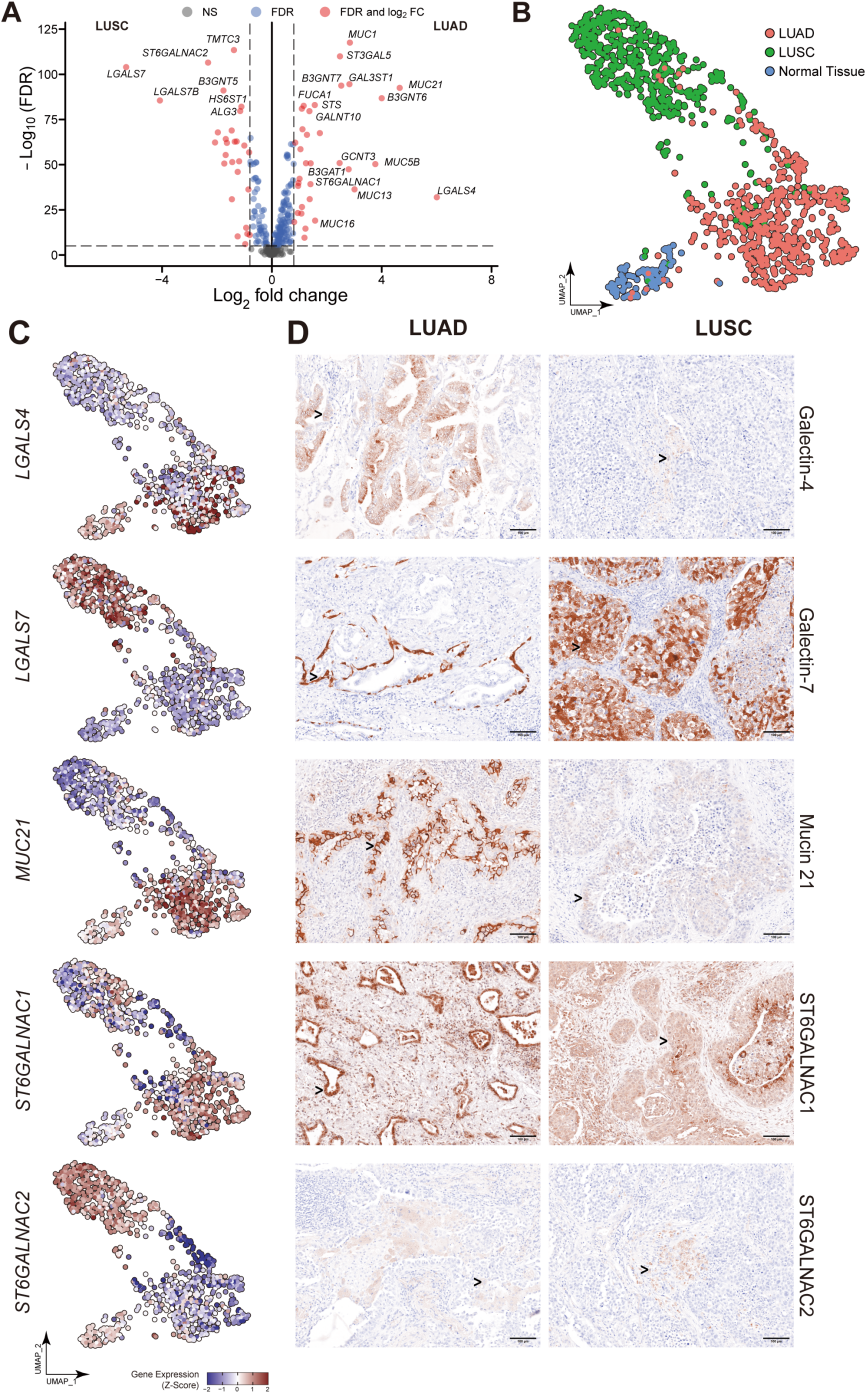
Essential glyco-relevant genes integrated from the TCGA dataset and investigated by IHC staining. **(A)** Differentially expressed genes (DEGs) associated with glycosylation between adenocarcinoma (LUAD) and squamous cell carcinoma (LUSC) were compared in the volcano plot (n=288 variables). **(B)** UMAP visualization of features from the TCGA dataset derived from adjacent normal, LUAD, and LUSC. **(C)** Feature plots of *LGALS4*, *LGALS7*, *MUC21*, *ST6GALNAC1*, and *ST6GALNAC2*. Upregulated expression is shown in muted red, and downregulated expression is in muted blue. **(D)** Immunohistochemical (IHC) staining of Galectin-4 (*LGALS4*), Galectin-7 (*LGALS7*), MUC21, ST6GALNAC1, and ST6GALNAC2 in FFPE tissue showed the expression difference between Stage 3 LUAD and LUSC patients (n=5 of each). Gene expression was scaled by z-score transformation. Genes with an absolute value of log_2_ fold change > 0.8 were screened out. False Discovery Rate (FDR) < 10–^5^ was considered statistically significant. The scale bar=100μm and arrows indicate individual cells with positive staining.

**Figure 4:**
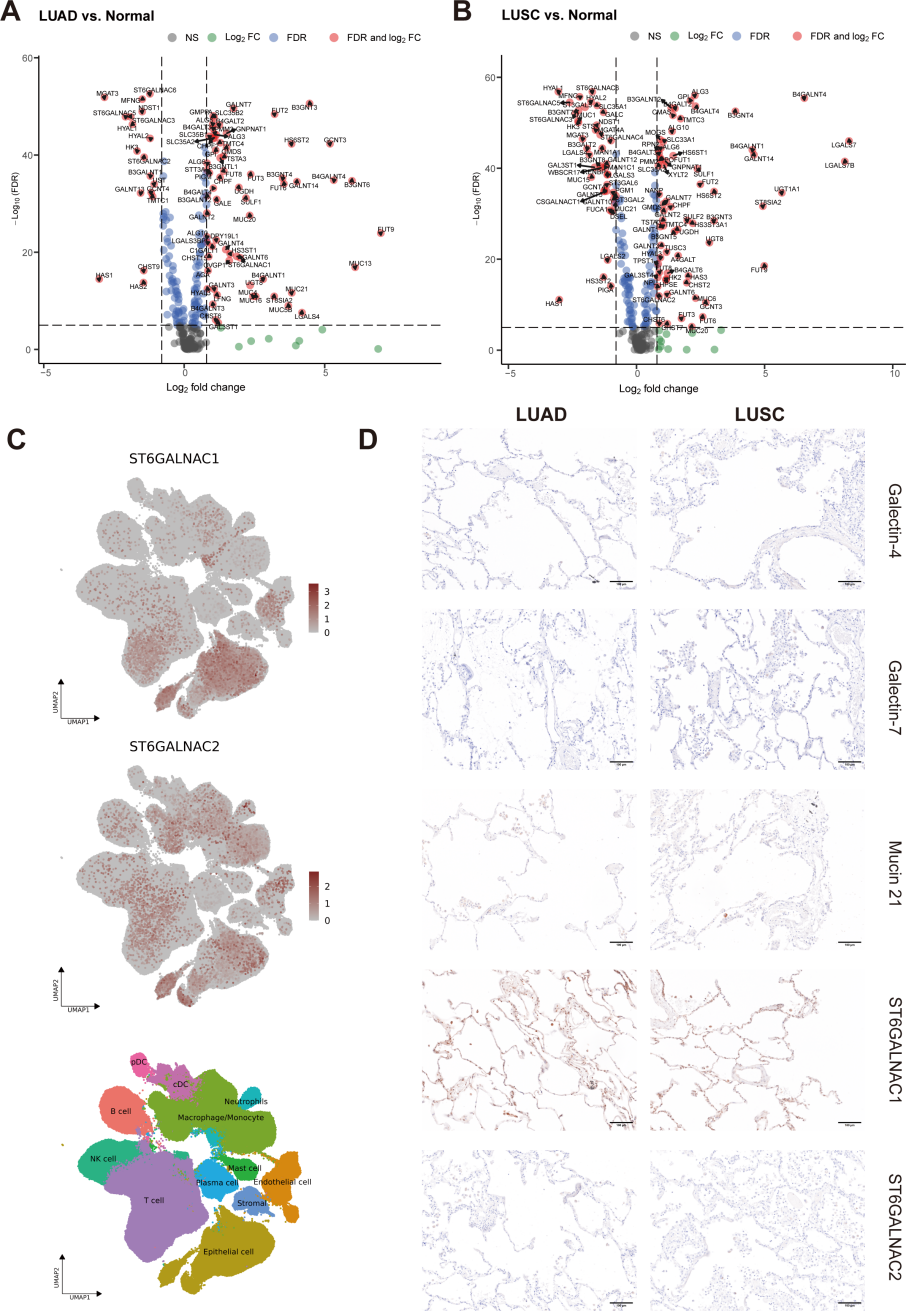
Identification of DEGs in TCGA dataset and essential gene signatures in scRNA-seq. **(A, B)** Related to **Figure 3A**. Volcano plot of glyco-associated DEGs of LUAD **(A)** and LUSC **(B)** compared to adjacent normal (n=292). The X-axis is log_2_ fold change, the dashed line was set to “|log_2_ fold change|=0.8”, the absolute value showed the multiple of the difference of genes, genes from the top left part were downregulated in the tumor, while upregulated genes were shown in the top right corner. The Y-axis is the negative base-10 logarithm of the False Discovery Rate (FDR), which increases with the increase of the significance of the difference, the horizontal dashed line was set to “-Log_10_(FDR)=5”. Significant genes calculated by log_2_ fold change and FDR were shown in red dots with gene names. **(C)** Related to **Figures 6A–C**. UMAP visualization of log-transformed and library-size corrected expression of *ST6GALNAC1* and *ST6GALNAC2* in the scRNA-seq dataset. **(D)** Related to **Figure 3D**. Immunohistochemical (IHC) staining of Galectin-4 (*LGALS4*), Galectin-7 (*LGALS7*), MUC21, ST6GALNAC1, and ST6GALNAC2 demonstrated their expression in non-malignant tissues adjacent to corresponding LUAD and LUSC samples. Gene expression was scaled by z-score transformation. The scale bar=100μm.

As our results showed that LUAD and LUSC present a dissimilar regulation of glycosylation-related genes when compared to adjacent normal samples, we next investigated further the differences between these 2 main subtypes of NSCLC (**Figure 3A**). We observed that high expression of *LGALS4*, *MUC21*, *B3GNT6*, *MUC5B*, *MUC13*, *MUC1*, *GAL3ST1*, *B3GAT1*, *B3GNT7*, and *ST3GAL5* was associated with LUAD, while high expression of *LGALS7*, *LGALS7B*, *ST6GALNAC2*, *B3GNT5*, *HS6ST1*, *TMTC3*, and *ALG3* was associated with LUSC. In **Figures 4A, B**, we also compared DEGs from LUAD and LUSC versus adjacent normal tissue, respectively. In LUAD, *FUT9*, *MUC13*, *B3GNT6*, *B4GALNT4*, *GCNT3*, *B3GNT3*, *LGALS4*, *GALNT14*, *MUC21*, and *HS6ST2* were found as top 10 most highly overexpressed genes. In LUSC, the top 10 most highly overexpressed genes were *LGALS7*, *LGALS7B*, *B4GALNT4*, *UGT1A1*, *FUT9*, *ST8SIA2*, *GALNT14*, *B4GALNT1*, *B3GNT4*, and *HS6ST2*.

**Figure 5:**
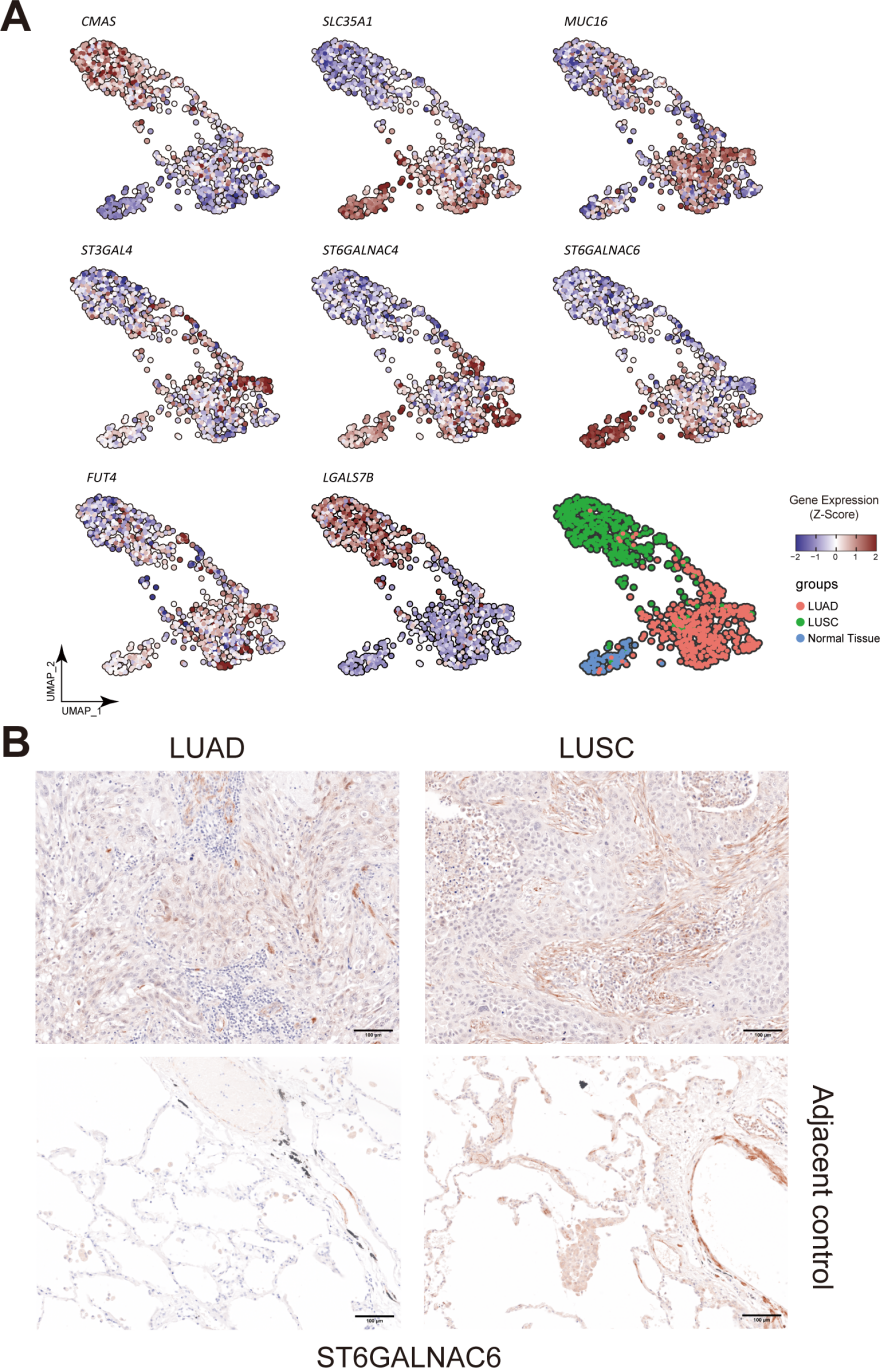
Landscape of different glyco-associated genes integrated from the TCGA dataset. **(A)** Related to **Figure 3C**. Feature plots show the expression of *CMAS*, *SLC35A1*, *MUC16*, *FUT4*, *ST3GAL4*, *ST6GALNAC4*, *ST6GALNAC6*, and *LGALS7B* in the TCGA dataset. Upregulated expression is shown in muted red, and downregulated expression is in muted blue. **(B)** Immunohistochemical **(IHC)** staining of ST6GALNAC6 in FFPE tissue showed the expression difference between Stage 3 LUAD and LUSC patients (n=5 of each). The second row showed ST6GALNAC6 expression in the corresponding adjacent non-malignant tissues. Gene expression was scaled by z-score transformation.

After reducing dimensions of UMAP, adjacent normal, LUAD, and LUSC were clustered into different groups based on their biological properties (**Figure 3B**). Then we selected a few significant DEGs from the comparison of LUAD and LUSC and subjected them to further analysis (*LGALS4*, *LGALS7*, *LGALS7B*, *MUC21*, *ST6GALNAC1* and *ST6GALNAC2*). In the UMAP plots shown in **Figure 3C**, *LGALS4* and *MUC21* were consistently found to be highly expressed in LUAD, whereas they were downregulated in LUSC compared to the control group. *ST6GALNAC1* exhibited high expression levels in LUAD, with less expression in the LUSC cluster. In contrast, we detected higher levels of *LGALS7*, *LGALS7B*, and *ST6GALNAC2* expression in LUSC than in LUAD (**Figures 3C**, **5A**).

To evaluate the differential expression of *LGALS4*, *LGALS7*, *MUC21*, *ST6GALNAC1*, and *ST6GALNAC2* in human tissue, IHC staining was employed in both LUSC and LUAD subtypes of lung cancer. As shown in **Figure 3D**, results confirm that Galectin-4 (*LGALS4*) is highly expressed in epithelial cells of LUAD, while Galectin-7 (*LGALS7*) is highly expressed in epithelial cells of LUSC. MUC21 exhibits high expression in the epithelial cells of LUAD, contrasting with dim staining observed in LUSC. ST6GALNAC1 demonstrates high expression in the epithelial cells of LUAD compared to LUSC. Furthermore, it was weakly stained in the stroma area of LUAD and LUSC tissue. For ST6GALNAC2, we discovered that it was also weakly stained in the stroma region, although there were no appreciable variations between LUAD and LUSC. As shown in **Figure 4D**, adjacent non-malignant tissues exhibited absent or markedly weaker expression for Galectin-4, Galectin-7, MUC21, ST6GALNAC1, and ST6GALNAC2 compared to the corresponding cancer tissues.

### Contribution of the malignant cell compartment to glyco-associated genes

**Figure 6:**
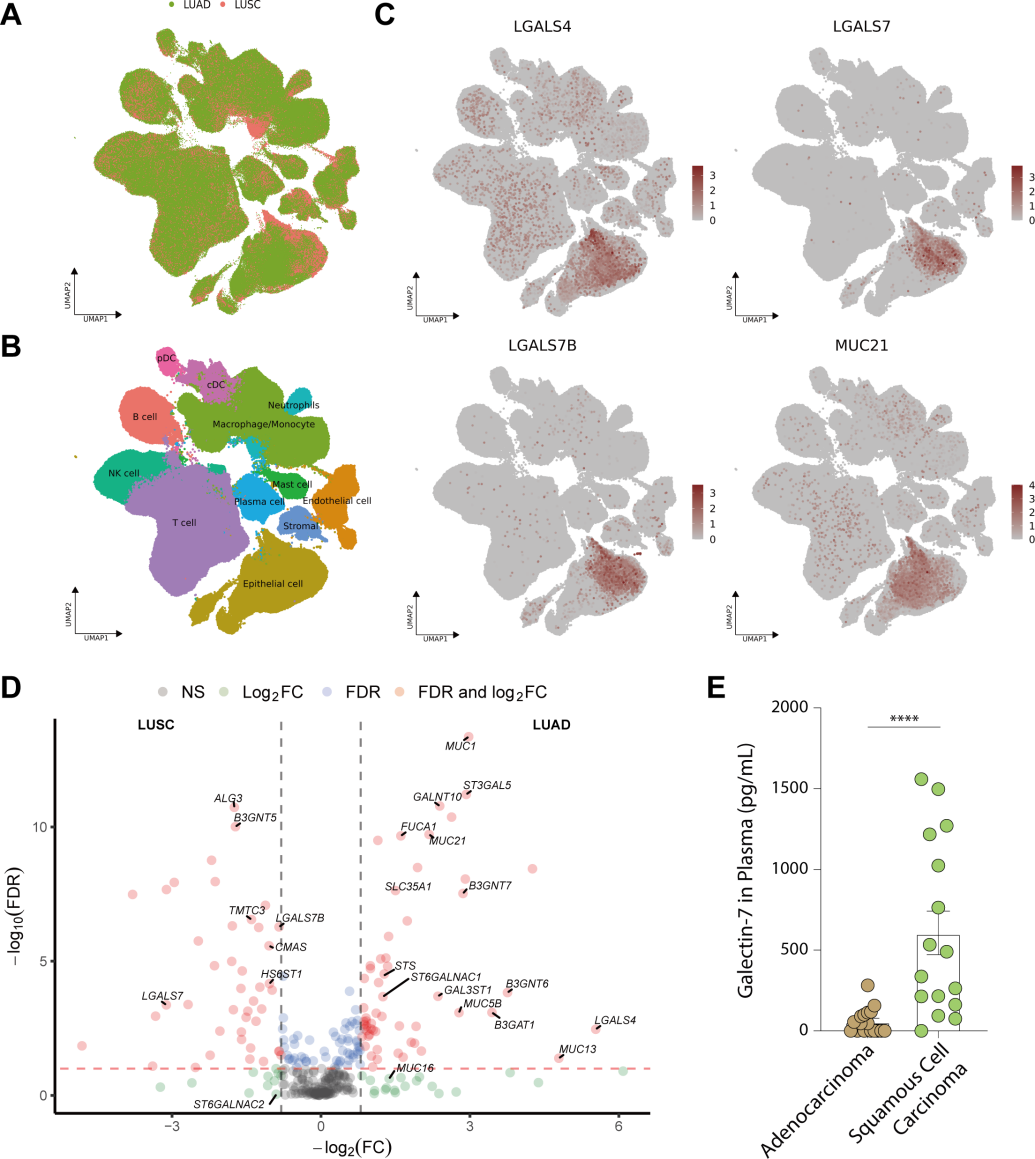
Expression of essential glyco-relevant genes in scRNA-seq dataset and quantification of Galectin-7 in plasma of lung cancer patients. **(A-B)** UMAP visualization of all features annotated by histological subtype (LUAD or LUSC, A) and 12 major cell types **(B)**. **(C)** UMAP visualization of log-transformed and library-size corrected expression of LUAD-related genes (*LGALS4*, *MUC21*) and LUSC-related genes (*LGALS7*, *LGALS7B*). **(D)** In the volcano plot, DEGs associated with glycosylation between LUAD and LUSC were compared in pseudobulk mixtures generated from the malignant cell compartments. **(E)** Plasma concentration of Galectin-7 in adenocarcinoma (n = 20) and squamous cell carcinoma (n = 16) of the lung. Genes with an absolute value of log_2_ fold change > 0.8 were screened out. False Discovery Rate (FDR) < 10^-2^ was considered statistically significant. ****, P<0.0001.

In order to further explore the mRNA expression of these candidate genes in NSCLC, we examined Single-cell RNA-seq (scRNA-seq) data of 345,260 cells from 163 LUAD patients and 128,423 cells from LUSC patients retrieved from Salcher et al. (11) (**Figure 6A**). The scRNA-seq atlas consisted of 12 major cell types: epithelial cells, stromal cells, endothelial cells, macrophages/monocytes, T cells, natural killer (NK) cells, neutrophils, B cells, plasma cells, plasmacytoid DCs (pDCs), conventional or classical DCs (cDCs) and mast cells (**Figure 6B**). From all annotations of various cell types, we found that the epithelial cell component of LUAD expressed more *LGALS4* and *MUC21* than that of LUSC (**Figure 6C**). In contrast, there was increased expression of *LGALS7* and *LGALS7B* in LUSC. Furthermore, in the tumor microenvironment of NSCLC, *ST6GALNAC1* and *ST6GALNAC2* showed elevated expression levels not only in epithelial cells but also in T cells and macrophages/monocytes (**Figure 4C**).

Since malignant epithelial cells were the main component that contributes to the signature of *LGALS4*, *LGALS7*, *MUC21*, and *ST6GALNAC1*, we further analyzed DEGs in epithelial cells of Single-cell data (**Figure 6D**). Our analysis revealed a consistent trend with our findings from TCGA data, showing higher expression of *LGALS4*, *MUC21*, and *ST6GALNAC1* in LUAD compared to LUSC, while *LGALS7* and *LGALS7B* exhibited elevated expression in LUSC compared to LUAD. These results further validate our exploration from tissue staining.

### Comparison of Galectin-7 expression in plasma of LUAD and LUSC patients

Furthermore, we wanted to confirm that Galectin-7 expression can be used to distinguish LUSC from LUAD. Since Galectins can be secreted, we set out to detect its presence in serum of LUSC and LUAD patients. An ELISA kit was used to detect the secretion level of Galectin-7 in the plasma of lung cancer patients (Adenocarcinoma (n=20) vs. Squamous cell carcinoma (n=16)). We measured significantly higher levels of Galectin-7 in plasma from squamous cell carcinoma patients than in plasma from adenocarcinoma patients, suggesting that it could serve as a biomarker for LUSC (**Figure 6E**, P < 0.0001).

### Analysis of potential prognostic value of glyco-associated gene expression in NSCLC

**Figure 7:**
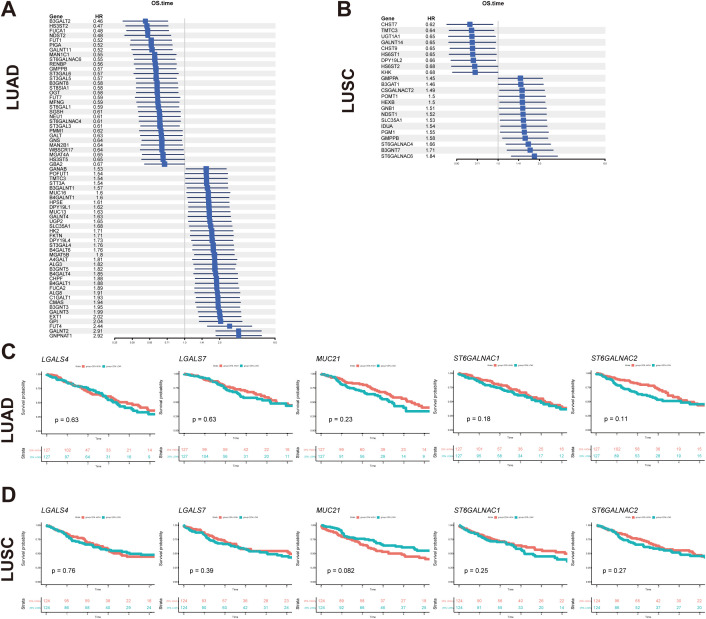
Glyco-related prognostic gene exploration using TCGA dataset. **(A, B)** Forest map showed glycol-associated DEGs of LUAD **(A)** and LUSC **(B)** in the univariate Cox regression model for overall survival time. **(C, D)** The survival analysis of key glyco-associated genes (*LGALS4*, *LGALS7*, *MUC21*, *ST6GALNAC1*, and *ST6GALNAC2*) was shown in Kaplan-Meier plots of LUAD and LUSC. The top 25% with high expression and the bottom 25% with low expression for each gene are separated into different subgroups. *The p-value* of survival analysis is based on the log-rank test.

**Figure 8:**
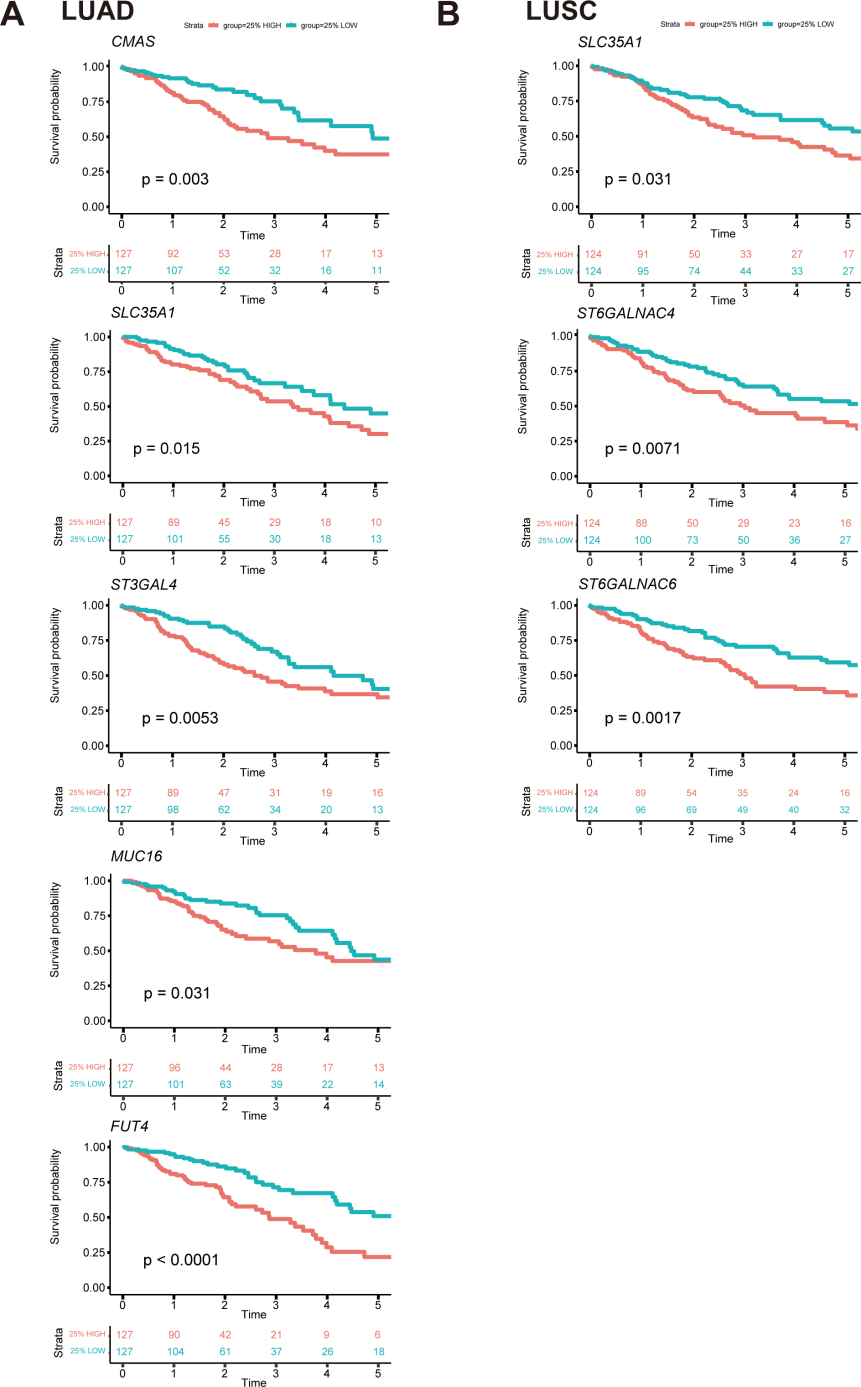
Survival curves of essential genes related to prognosis among all glyco-relevant genes. **(A)** Kaplan-Meier (KM) plot shows the overall survival difference of *CMAS*, *SLC35A1*, *ST3GAL4*, *MUC16*, and *FUT4* across LUAD patients. **(B)** KM plot shows the overall survival difference of *SLC35A1*, *ST6GALNAC4*, and *ST6GALNAC6* across LUSC patients. The top 25% with high expression and the bottom 25% with low expression for each gene are separated into different subgroups. *The p-value* of survival analysis is based on the log-rank test.

Univariate Cox regression analysis was conducted to evaluate the survival implications of all glyco-associated genes in LUAD based on Hazard ratio values (**Figure 7A**). Our findings revealed that elevated expression of 36 genes was linked to unfavorable survival outcomes, whereas 31 genes were correlated with better survival outcomes. Patients were divided into two groups based on the expression level of each gene, specifically the top 25% with high expression and the bottom 25% with low expression. Subsequently, survival curves were plotted, comparing the impact of individual genes on survival. Notably, *CMAS*, *SLC35A1*, *ST3GAL4*, *MUC16*, and *FUT4* were identified as genes linked to poor survival (**Figure 8A**).

In parallel, the survival implications of all glyco-associated genes were also assessed in LUSC. We found that 14 genes with high expression were associated with bad survival of squamous cell carcinoma, and 9 genes were associated with better survival (**Figure 7B**). *SLC35A1*, *ST6GALNAC4*, and *ST6GALNAC6* were selected and validated, demonstrating that their elevated expression is associated with poor survival of LUSC patients (**Figure 8B**). No significant differences in survival were observed in LUAD and LUSC for high and low expression of *LGALS4*, *LGALS7*, *MUC21*, *ST6GALNAC1*, and *ST6GALNAC2* (**Figures 7C, D**).

UMAP projection was utilized to visualize features of these seven survival-relevant genes in both LUAD and LUSC (**Figure 5A**). *SLC35A1*, *MUC16*, *FUT4*, *ST3GAL4*, *ST6GALNAC4*, and *ST6GALNAC6* showed more expression in LUAD than in LUSC (**Supplementary Table S4**). In contrast, *CMAS* and *LGALS7B* were highly expressed in LUSC.

Intriguingly, it was also observed that the expression of *ST6GALNAC6* in the adjacent normal cluster was higher than that in the clusters of LUAD and LUSC; and *SLC35A1* was also highly expressed in adjacent normal tissue (**Figure 5A**). Furthermore, tissue staining revealed that *ST6GALNAC6* expression levels were comparable between LUAD and LUSC, and neither subtype showed significant differences relative to adjacent non-malignant tissues (**Figure 5B**). High *ST6GALNAC6* expression in LUSC was associated with bad survival, while the opposite was observed in LUAD (**Figures 8B**, **7A, B**).

## Discussion

Our study presents the first comprehensive analysis of expression variations across the entire glycosylation-associated gene atlas in NSCLC, aiming to identify markers that correlate with survival. We found specific genes for mucins, galectins, and members of the ST6GALNAC sialyltransferase family, capable of distinguishing between LUSC and LUAD, which are the two major histological subtypes of NSCLC.

### *CMAS* and *SLC35A1*

We found that *CMAS* and *SLC35A1* correlated with worse survival outcomes in lung adenocarcinoma. The activity of *CMAS*, responsible for converting Neu5Ac to CMP-Neu5Ac, has been described to be significantly associated with decreased survival of breast cancer (13). Previously, we found that *CMAS* KO in a murine model resulted in enhanced infiltration of CD4^+^ and CD8^+^ T cells within the tumor microenvironment of pancreatic ductal carcinoma and improved survival outcomes (14). In contrast, upregulation of *CMAS* in pancreatic tumors increased sialylation and promoted immune suppression (9, 15).

Additionally, SLC35A1 transports cytidine 5’ monophosphate (CMP)-sialic acid, the donor substrate for a range of sialyltransferases, thereby modulating sialylation within the Golgi apparatus (16). Notably, *SLC35A1* was also seen to be associated with worse survival outcomes in lung squamous cell carcinoma. *SLC35A1* knock-down in B16 melanoma reduced tumor growth due to the reduction of sialylation and enhanced effector T cell response (17). These studies demonstrated that the reduction of sialylation lowers the engagement of Siglecs (Sialic acid-binding lectin receptors) and their immune inhibitory function; and showed that sialylation as glyco-immune checkpoint modulates tumor growth (18).

### *MUC1*, *MUC16*, and *MUC21*

The mucin family comprises a large group of heavily O-glycosylated proteins, classified into membrane-bound and secretory types. Among membrane-bound mucins, MUC1, MUC16, and MUC21 attach to cell surfaces via their transmembrane domains (19). In lung cancer, high-grade polarized expression of MUC1 is observed in well-differentiated adenocarcinoma, while depolarized MUC1—extending from the apex to the entire surface—is associated with advanced stages, lymph node metastasis, and disruption of cell–cell and cell–matrix interactions (20, 21). MUC16 was overexpressed in both human primary lung carcinoma and associated lymph node metastases, potentially playing a role in the epithelial-to-mesenchymal transition during lung cancer cell metastasis (22). In addition, the highly glycosylated tandem repeat domain of MUC21 on the cell surface impairs cell–cell and cell–matrix adhesion via steric hindrance, potentially contributing to tumor metastasis through enhanced cell migration and invasion (23).

Our results on mRNA differential expression, both in bulk data and in malignant cells from the scRNA-seq data, demonstrated that *MUC1* and *MUC16* were substantially overexpressed in LUAD compared to LUSC, with *MUC1*’s expression validated by IHC (24). Retrospective studies align these findings with poor survival in NSCLC, though our data did not confirm *MUC1*’s correlation with poor survival, despite previous affirmations (24–26). *MUC21* showed higher mRNA expression in LUAD than in adjacent normal or LUSC tissues, consistent with GEPIA database findings (26). IHC revealed greater *MUC21* expression in certain cancer cell patterns, particularly micropapillary, papillary, and lepidic, compared to cohesive tumor components in LUAD patients (27).

An increasing number of clinical trials have focused on targeting mucins such as MUC1, employing modalities including monoclonal antibodies (e.g., PankoMab-GEX for TA-MUC1), liposomal vaccines (e.g., tecemotide), and CAR-T cell therapy, demonstrating clinical feasibility of MUC1-targeted therapy (28, 29). MUC21 is highly expressed in micropapillary structures and may contribute to the transition from pure lepidic to micropapillary pattern, suggesting its involvement in LUAD progression and potential as a biomarker for predicting disease progression (30). Specific glycosylated forms of MUC21 may contribute to the development of LUAD with EGFR mutations, which are strongly associated with a dominant micropapillary growth pattern (30, 31). Therapeutic strategies targeting MUC21 may offer a promising approach, particularly in LUAD subtypes characterized by micropapillary architecture and EGFR mutations, where its expression is implicated in tumor progression.

### Galectin-4 and Galectin-7

Galectins, a family of carbohydrate-binding proteins, are classified into prototypical, tandem-repeat, and chimeric types based on their carbohydrate recognition domains (CRDs) (32). These proteins serve as diagnostic biomarkers for detecting malignant tumors (33). Among the galectins that emerged as most relevant in our research, Galectin-2 and Galectin-7 are prototypical galectins, Galectin-4 is a kind of tandem−repeat galectin, and Galectin−3 is the only chimeric galectin, which contains a single CRD and a large amino−terminal domain (32).

Galectin-4 was strongly expressed in LUAD patients with lymph node metastasis and was associated with aggressive cancer traits such as lymphatic and venous invasion, although it did not correlate with overall or recurrence-free survival (34).

Conversely, Galectin-7, significantly upregulated in lung squamous cell carcinoma (LUSC) versus LUAD, is validated by increased serum levels and higher immunohistochemistry (IHC) expression in LUSC, indicating its potential as a biomarker. In a syngeneic mouse squamous cell carcinoma (SCC) model, Galectin-7 has been recognized as a mediator of metastasis linked to immunosuppression, exhibiting significant induction in the tumor microenvironment during tumorigenesis, and is released extracellularly at advanced stages of tumor growth (35). Consistent with this, a previous study of breast carcinoma has demonstrated that Galectin-7, absent in low-grade but upregulated in high-grade, is associated with increased metastasis to the lungs and bones (36).

### *ST6GALNAC1*, *ST6GALNAC2*, *ST6GALNAC4* and *ST6GALNAC6*

Enzymes from the ST6GALNAC sialyltransferase family are involved in α2,6-sialylation on glycolipids and O-glycosylated proteins, via the addition of sialic acid to GalNAc residue (5). Specifically, *ST6GALNAC1* and *ST6GALNAC2* have been shown to enhance metastatic potential in various cancers (37, 38).

High expression of *ST6GALNAC1* induces the synthesis of the sialyl-Tn antigen via α2,6-linkage by promoting the sialylation of MUC5AC, thereby facilitating liver metastasis in LUAD (37). It shows high expression in LUAD with prominent localization on tumor cell membranes, distinguishing it from poorly differentiated squamous cell carcinoma (PDSCC) (39).

*ST6GALNAC2* mainly sialylates T antigens, contributing to the formation of disialyl-T antigen. ST6GALNAC2 expression pattern and its potential implications in human lung cancer tissues remain unexplored in prior research. Notably, in other studies, disialyl-T antigen has been defined as a ligand for Siglec-7, an immune-inhibitory glycan-binding receptor expressed on NK cells and myeloid cells, which suppresses immune function upon ligand engagement (40). In addition, ST6GALNAC2 impacts Galectin-3 binding, with high expression correlating with reduced lung metastasis and improved survival in ER- breast cancers (38). Furthermore, ST6GALNAC2 has been shown to promote the invasive capabilities of breast carcinoma cells, potentially through activation of the PI3K/Akt/NF-κB signaling pathway (41). In contrast, elevated expression of *ST6GALNAC2* in colorectal cancer is linked to poorer survival outcomes (42).

Based on our existing scRNA-seq data, T cells and macrophages/monocytes are prominent contributors to higher *ST6GALNAC2* mRNA expression, which further supported our IHC staining result. This fact might help to explain our findings that it affected the analysis result of bulk data, and did not significantly differentiate between LUAD and LUSC in the malignant cell compartment.

*ST6GALNAC4* is involved in the synthesis of disialyl-T antigen from sialyl-T antigen and GD1α from GM1b (43). *ST6GALNAC4* is tied to adverse outcomes in LUSC per TCGA data. It increases T antigen expression and Galectin-3+ macrophage recruitment, which supports tumor invasion and immune suppression (44–46). It could be interpreted that in the absence of core 2 O-glycans, the increase of disialyl-T antigen portion is facilitated by *ST6GALNAC4* prevents glycan elongation on the cell surface. Consequently, this situation mediates the adhesion of Galectin-3 to interact with residual T antigens (43).

*ST6GALNAC6* is involved in the synthesis of ganglioside GD1α (representative 0-series gangliosides), GT1aα (a-series), and GQ1bα (b-series) from GM1b, GD1a, and GT1b, respectively (47). Downregulation of *ST6GALNAC6* mRNA was detected in human colon cancer compared with non-malignant epithelium, accompanied by a concomitant decrease in disialyl-Lewis^a^ and an increase in sialyl Lewis^a^ during malignant transformation (48). Another discovery from a renal cancer study revealed that silencing *ST6GALNAC6* in cancer cells resulted in a reduction of metastatic ability (49). Despite notable expression differences in non-malignant versus malignant lung tissues, the molecular mechanisms linking *ST6GALNAC4* and *ST6GALNAC6* to clinical outcomes in NSCLC remain underexplored.

### From CEA to novel glyco-signatures: promise and challenges

Carcinoembryonic antigen (CEA) is an acidic glycoprotein associated with human embryonic antigen, characterized by extensive N-glycosylation, and has been utilized as a diagnostic or prognostic marker in various cancer types, including lung cancer (50–52). However, its clinical utility is limited by relatively low specificity, making it more suitable for disease monitoring rather than early detection. In contrast, our study identifies several glycosylation-associated genes with potential diagnostic value in NSCLC. These novel biomarkers may offer improved tumor subtype discrimination. Further functional validation and clinical correlation studies are warranted to elucidate their potential utility in the diagnosis and therapeutic intervention of lung cancer.

Although glycosylation plays a crucial biological role, its study remains challenging due to the structural complexity and dynamic biosynthesis of the glycan chain, as well as the lack of a direct structure–function relationship (53). Furthermore, the intrinsic heterogeneity of tumor tissues adds another layer of difficulty in translating glycosylation-based findings into effective clinical therapies.

## Conclusions

In this study, we have identified several glyco-associated biomarkers that have the potential to be used in diagnostic applications, including Galectin-4, Galectin-7, MUC21, ST6GALNAC1, and ST6GALNAC2. Also, these 5 genes, which are mostly produced by malignant cell compartments in the TME, could serve as biomarkers for differentiating between LUSC and LUAD. Galectin-7 could serve in clinical plasma detection, of which the result combined with pathological classification could improve patient identification.

## Data Availability

The original contributions presented in the study are included in the article/**Supplementary Material**. Further inquiries can be directed to the corresponding author.

## Supplementary material

Supplementary Table 1 Antibodies for tissue staining.

Supplementary Table 2 Differentially expressed glyco-genes in TCGA (LUAD vs. normal).

Supplementary Table 3 Differentially expressed glyco-genes in TCGA (LUSC vs. normal).

Supplementary Table 4 Differentially expressed glyco-genes in TCGA (LUAD vs. LUSC).

Supplementary Table 5 Key clinical characteristics of the patient cohort.

## References

[1] Bray F Ferlay J Soerjomataram I Siegel RL Torre LA Jemal A. Global cancer statistics 2018: globocan estimates of incidence and mortality worldwide for 36 cancers in 185 countries. CA: Cancer J Clin. (2018) 68:394–424. doi: 10.3322/caac.21492, PMID: , PMID: 30207593

[2] Silsirivanit A. Glycosylation markers in cancer. Adv Clin Chem. (2019) 89:189–213. doi: 10.1016/bs.acc.2018.12.005, PMID: , PMID: 30797469

[3] Fu C Zhao H Wang Y Cai H Xiao Y Zeng Y. Tumor-associated antigens: tn antigen, stn antigen, and T antigen. Hla. (2016) 88:275–86. doi: 10.1111/tan.12900, PMID: , PMID: 27679419

[4] Wi DH Cha JH Jung YS. Mucin in cancer: A stealth cloak for cancer cells. BMB Rep. (2021) 54:344–55. doi: 10.5483/BMBRep.2021.54.7.064, PMID: , PMID: 34154702 PMC8328826

[5] Hugonnet M Singh P Haas Q von Gunten S. The distinct roles of sialyltransferases in cancer biology and onco-immunology. Front Immunol. (2021) 12:799861. doi: 10.3389/fimmu.2021.799861, PMID: , PMID: 34975914 PMC8718907

[6] Hsu YL Wu CY Hung JY Lin YS Huang MS Kuo PL. Galectin-1 promotes lung cancer tumor metastasis by potentiating integrin α6β4 and notch1/jagged2 signaling pathway. Carcinogenesis. (2013) 34:1370–81. doi: 10.1093/carcin/bgt040, PMID: , PMID: 23389289

[7] Dobie C Skropeta D. Insights into the role of sialylation in cancer progression and metastasis. Br J Cancer. (2021) 124:76–90. doi: 10.1038/s41416-020-01126-7, PMID: , PMID: 33144696 PMC7782833

[8] Paulson JC Rademacher C. Glycan terminator. Nat Struct Mol Biol. (2009) 16:1121–2. doi: 10.1038/nsmb1109-1121, PMID: , PMID: 19888308 PMC2838401

[9] Rodriguez E Boelaars K Brown K Eveline Li RJ Kruijssen L Bruijns SCM. Sialic acids in pancreatic cancer cells drive tumour-associated macrophage differentiation via the siglec receptors siglec-7 and siglec-9. Nat Commun. (2021) 12:1270. doi: 10.1038/s41467-021-21550-4, PMID: , PMID: 33627655 PMC7904912

[10] Rodriguez E Lindijer DV van Vliet SJ Garcia Vallejo JJ van Kooyk Y. The transcriptional landscape of glycosylation-related genes in cancer. iScience. (2024) 27:109037. doi: 10.1016/j.isci.2024.109037, PMID: , PMID: 38384845 PMC10879703

[11] Salcher S Sturm G Horvath L Untergasser G Kuempers C Fotakis G. High-resolution single-cell atlas reveals diversity and plasticity of tissue-resident neutrophils in non-small cell lung cancer. Cancer Cell. (2022) 40:1503–20.e8. doi: 10.1016/j.ccell.2022.10.008, PMID: , PMID: 36368318 PMC9767679

[12] Squair JW Gautier M Kathe C Anderson MA James ND Hutson TH. Confronting false discoveries in single-cell differential expression. Nat Commun. (2021) 12:5692. doi: 10.1038/s41467-021-25960-2, PMID: , PMID: 34584091 PMC8479118

[13] Teoh ST Ogrodzinski MP Ross C Hunter KW Lunt SY. Sialic acid metabolism: A key player in breast cancer metastasis revealed by metabolomics. Front Oncol. (2018) 8:174. doi: 10.3389/fonc.2018.00174, PMID: , PMID: 29892572 PMC5985449

[14] Boelaars K Goossens-Kruijssen L Wang D de Winde CM Rodriguez E Lindijer D. Unraveling the impact of sialic acids on the immune landscape and immunotherapy efficacy in pancreatic cancer. J immunotherapy Cancer. (2023) 11(11). doi: 10.1136/jitc-2023-007805, PMID: , PMID: 37940346 PMC10632901

[15] Boelaars K Rodriguez E Huinen ZR Liu C Wang D Springer BO. Pancreatic cancer-associated fibroblasts modulate macrophage differentiation via sialic acid-siglec interactions. Commun Biol. (2024) 7:430. doi: 10.1038/s42003-024-06087-8, PMID: , PMID: 38594506 PMC11003967

[16] Ury B Potelle S Caligiore F Whorton MR Bommer GT. The promiscuous binding pocket of slc35a1 ensures redundant transport of cdp-ribitol to the golgi. J Biol Chem. (2021) 296:100789. doi: 10.1016/j.jbc.2021.100789, PMID: , PMID: 34015330 PMC8192872

[17] Perdicchio M Cornelissen LA Streng-Ouwehand I Engels S Verstege MI Boon L. Tumor sialylation impedes T cell mediated anti-tumor responses while promoting tumor associated-regulatory T cells. Oncotarget. (2016) 7:8771–82. doi: 10.18632/oncotarget.6822, PMID: , PMID: 26741508 PMC4891003

[18] RodrÍguez E Schetters STT van Kooyk Y. The tumour glyco-code as a novel immune checkpoint for immunotherapy. Nat Rev Immunol. (2018) 18:204–11. doi: 10.1038/nri.2018.3, PMID: , PMID: 29398707

[19] Hattrup CL Gendler SJ. Structure and function of the cell surface (Tethered) mucins. Annu Rev Physiol. (2008) 70:431–57. doi: 10.1146/annurev.physiol.70.113006.100659, PMID: , PMID: 17850209

[20] Jarrard JA Linnoila RI Lee H Steinberg SM Witschi H Szabo E. Muc1 is a novel marker for the type ii pneumocyte lineage during lung carcinogenesis. Cancer Res. (1998) 58:5582–9., PMID: , PMID: 9850098

[21] Guddo F Giatromanolaki A Patriarca C Hilkens J Reina C Alfano RM. Depolarized expression of episialin (Ema, muc1) in lung adenocarcinoma is associated with tumor progression. Anticancer Res. (1998) 18:1915–20., PMID: , PMID: 9677444

[22] Lakshmanan I Salfity S Seshacharyulu P Rachagani S Thomas A Das S. Muc16 regulates tspyl5 for lung cancer cell growth and chemoresistance by suppressing P53. Clin Cancer research: an Off J Am Assoc Cancer Res. (2017) 23:3906–17. doi: 10.1158/1078-0432.Ccr-16-2530, PMID: , PMID: 28196872 PMC5511558

[23] Yi Y Kamata-Sakurai M Denda-Nagai K Itoh T Okada K Ishii-Schrade K. Mucin 21/epiglycanin modulates cell adhesion. J Biol Chem. (2010) 285:21233–40. doi: 10.1074/jbc.M109.082875, PMID: , PMID: 20388707 PMC2898422

[24] Situ D Wang J Ma Y Zhu Z Hu Y Long H. Expression and prognostic relevance of muc1 in stage ib non-small cell lung cancer. Med Oncol (Northwood London England). (2011) 28 Suppl 1:S596–604. doi: 10.1007/s12032-010-9752-4, PMID: , PMID: 21116877

[25] Xie Q Zhao S Liu W Cui Y Li F Li Z. Ybx1 enhances metastasis and stemness by transcriptionally regulating muc1 in lung adenocarcinoma. Front Oncol. (2021) 11:702491. doi: 10.3389/fonc.2021.702491, PMID: , PMID: 34976785 PMC8714800

[26] Tu J Tang M Li G Chen L Wang Y Huang Y. Expression of mucin family proteins in non-small-cell lung cancer and its role in evaluation of prognosis. J Oncol. (2022) 2022:4181658. doi: 10.1155/2022/4181658, PMID: , PMID: 36059804 PMC9439898

[27] Yoshimoto T Matsubara D Soda M Ueno T Amano Y Kihara A. Mucin 21 is a key molecule involved in the incohesive growth pattern in lung adenocarcinoma. Cancer Sci. (2019) 110:3006–11. doi: 10.1111/cas.14129, PMID: , PMID: 31301084 PMC6726699

[28] Fiedler W DeDosso S Cresta S Weidmann J Tessari A Salzberg M. A phase I study of pankomab-gex, a humanised glyco-optimised monoclonal antibody to a novel tumour-specific muc1 glycopeptide epitope in patients with advanced carcinomas. Eur J Cancer (Oxford England: 1990). (2016) 63:55–63. doi: 10.1016/j.ejca.2016.05.003, PMID: , PMID: 27285281

[29] Butts C Socinski MA Mitchell PL Thatcher N Havel L Krzakowski M. Tecemotide (L-blp25) versus placebo after chemoradiotherapy for stage iii non-small-cell lung cancer (Start): A randomised, double-blind, phase 3 trial. Lancet Oncol. (2014) 15:59–68. doi: 10.1016/s1470-2045(13)70510-2, PMID: , PMID: 24331154

[30] Matsumura M Okudela K Nakashima Y Mitsui H Denda-Nagai K Suzuki T. Specific expression of muc21 in micropapillary elements of lung adenocarcinomas - implications for the progression of egfr-mutated lung adenocarcinomas. PloS One. (2019) 14:e0215237. doi: 10.1371/journal.pone.0215237, PMID: , PMID: 30973916 PMC6459478

[31] Chao L Yi-Sheng H Yu C Li-Xu Y Xin-Lan L Dong-Lan L. Relevance of egfr mutation with micropapillary pattern according to the novel iaslc/ats/ers lung adenocarcinoma classification and correlation with prognosis in chinese patients. Lung Cancer (Amsterdam Netherlands). (2014) 86:164–9. doi: 10.1016/j.lungcan.2014.08.018, PMID: , PMID: 25236981

[32] Chetry M Thapa S Hu X Song Y Zhang J Zhu H. The role of galectins in tumor progression, treatment and prognosis of gynecological cancers. J Cancer. (2018) 9:4742–55. doi: 10.7150/jca.23628, PMID: , PMID: 30588260 PMC6299382

[33] Chang WA Tsai MJ Kuo PL Hung JY. Role of galectins in lung cancer. Oncol Lett. (2017) 14:5077–84. doi: 10.3892/ol.2017.6882, PMID: , PMID: 29113148 PMC5662908

[34] Hayashi T Saito T Fujimura T Hara K Takamochi K Mitani K. Galectin-4, a novel predictor for lymph node metastasis in lung adenocarcinoma. PloS One. (2013) 8:e81883. doi: 10.1371/journal.pone.0081883, PMID: , PMID: 24339976 PMC3858289

[35] An J Nagaki Y Motoyama S Kuze Y Hoshizaki M Kemuriyama K. Identification of galectin-7 as a crucial metastatic enhancer of squamous cell carcinoma associated with immunosuppression. Oncogene. (2022) 41:5319–30. doi: 10.1038/s41388-022-02525-1, PMID: , PMID: 36335283

[36] Demers M Rose AA Grosset AA Biron-Pain K Gaboury L Siegel PM. Overexpression of galectin-7, a myoepithelial cell marker, enhances spontaneous metastasis of breast cancer cells. Am J Pathol. (2010) 176:3023–31. doi: 10.2353/ajpath.2010.090876, PMID: , PMID: 20382700 PMC2877862

[37] Lakshmanan I Chaudhary S Vengoji R Seshacharyulu P Rachagani S Carmicheal J. St6galnac-I promotes lung cancer metastasis by altering muc5ac sialylation. Mol Oncol. (2021) 15:1866–81. doi: 10.1002/1878-0261.12956, PMID: , PMID: 33792183 PMC8253099

[38] Murugaesu N Iravani M van Weverwijk A Ivetic A Johnson DA Antonopoulos A. An *in vivo* functional screen identifies st6galnac2 sialyltransferase as a breast cancer metastasis suppressor. Cancer Discov. (2014) 4:304–17. doi: 10.1158/2159-8290.Cd-13-0287, PMID: , PMID: 24520024

[39] Takamochi K Ohmiya H Itoh M Mogushi K Saito T Hara K. Novel biomarkers that assist in accurate discrimination of squamous cell carcinoma from adenocarcinoma of the lung. BMC Cancer. (2016) 16:760. doi: 10.1186/s12885-016-2792-1, PMID: , PMID: 27681076 PMC5041559

[40] Stewart N Daly J Drummond-Guy O Krishnamoorthy V Stark JC Riley NM. The glycoimmune checkpoint receptor siglec-7 interacts with T-cell ligands and regulates T-cell activation. J Biol Chem. (2024) 300:105579. doi: 10.1016/j.jbc.2023.105579, PMID: , PMID: 38141764 PMC10831161

[41] Ren D Jia L Li Y Gong Y Liu C Zhang X. St6galnacii mediates the invasive properties of breast carcinoma through pi3k/akt/nf-κb signaling pathway. IUBMB Life. (2014) 66:300–8. doi: 10.1002/iub.1268, PMID: , PMID: 24756995

[42] Schneider F Kemmner W Haensch W Franke G Gretschel S Karsten U. Overexpression of sialyltransferase cmp-sialic acid: galbeta1,3galnac-R alpha6-sialyltransferase is related to poor patient survival in human colorectal carcinomas. Cancer Res. (2001) 61:4605–11., PMID: , PMID: 11389097

[43] Dimitroff CJ. Galectin-binding O-glycosylations as regulators of Malignancy. Cancer Res. (2015) 75:3195–202. doi: 10.1158/0008-5472.Can-15-0834, PMID: , PMID: 26224120 PMC4537818

[44] Pucci M Duca M Malagolini N Dall’Olio F. Glycosyltransferases in cancer: prognostic biomarkers of survival in patient cohorts and impact on Malignancy in experimental models. Cancers (Basel). (2022) 14. doi: 10.3390/cancers14092128, PMID: , PMID: 35565254 PMC9100214

[45] Reticker-Flynn NE Bhatia SN. Aberrant glycosylation promotes lung cancer metastasis through adhesion to galectins in the metastatic niche. Cancer Discov. (2015) 5:168–81. doi: 10.1158/2159-8290.Cd-13-0760, PMID: , PMID: 25421439 PMC4367955

[46] Man D Jiang Y Zhang D Wu J Ding B Liu H. St6galnac4 promotes hepatocellular carcinogenesis by inducing abnormal glycosylation. J Trans Med. (2023) 21:420. doi: 10.1186/s12967-023-04191-7, PMID: , PMID: 37381011 PMC10308692

[47] Okajima T Chen HH Ito H Kiso M Tai T Furukawa K. Molecular cloning and expression of mouse gd1alpha/gt1aalpha/gq1balpha synthase (St6galnac vi) gene. J Biol Chem. (2000) 275:6717–23. doi: 10.1074/jbc.275.10.6717, PMID: , PMID: 10702226

[48] Miyazaki K Ohmori K Izawa M Koike T Kumamoto K Furukawa K. Loss of disialyl lewis(a), the ligand for lymphocyte inhibitory receptor sialic acid-binding immunoglobulin-like lectin-7 (Siglec-7) associated with increased sialyl lewis(a) expression on human colon cancers. Cancer Res. (2004) 64:4498–505. doi: 10.1158/0008-5472.Can-03-3614, PMID: , PMID: 15231659

[49] Kawasaki Y Ito A Kakoi N Shimada S Itoh J Mitsuzuka K. Ganglioside, disialosyl globopentaosylceramide (Dsgb5), enhances the migration of renal cell carcinoma cells. Tohoku J Exp Med. (2015) 236:1–7. doi: 10.1620/tjem.236.1, PMID: , PMID: 25864532

[50] Grunnet M Sorensen JB. Carcinoembryonic antigen (Cea) as tumor marker in lung cancer. Lung Cancer (Amsterdam Netherlands). (2012) 76:138–43. doi: 10.1016/j.lungcan.2011.11.012, PMID: , PMID: 22153832

[51] Hammarström S. The carcinoembryonic antigen (Cea) family: structures, suggested functions and expression in normal and Malignant tissues. Semin Cancer Biol. (1999) 9:67–81. doi: 10.1006/scbi.1998.0119, PMID: , PMID: 10202129

[52] Wang J Chu Y Li J Wang T Sun L Wang P. The clinical value of carcinoembryonic antigen for tumor metastasis assessment in lung cancer. PeerJ. (2019) 7:e7433. doi: 10.7717/peerj.7433, PMID: , PMID: 31410309 PMC6689222

[53] Xu X Peng Q Jiang X Tan S Yang W Han Y. Altered glycosylation in cancer: molecular functions and therapeutic potential. Cancer Commun (London England). (2024) 44:1316–36. doi: 10.1002/cac2.12610, PMID: , PMID: 39305520 PMC11570773

