# Supplementary material for: Analysis of cancer-associated glycosyltransferases reveals novel targets of non-small cell lung cancer pathogenesis
Source: Front Oncol. 2025 Jul 11;15:1601368. doi: 10.3389/fonc.2025.1601368 (PMC12290921; doi:10.3389/fonc.2025.1601368)
Supplement: Supplementary Table 1 — Antibodies for tissue staining. [file DataSheet1.docx]

# Table S1, Antibodies for tissue staining

| Antibodies | Order number | Manufacturer | Antigen retrieval buffer | Concentration |
| --- | --- | --- | --- | --- |
| Galectin4 | ab77995 | Abcam | Citrate | 1/200 |
| Galectin7 | ab108623 | Abcam | EDTA | 1/1500 |
| Mucin21 | With courtesy of Tatsuro Irimura from Juntendo University, Tokyo, Japan [23] | | None | 1/200 |
| ST6GALNAC1 | HPA014975 | Sigma-Aldrich | EDTA | 1/200 |
| ST6GALNAC2 | PA5-31353 | Thermo Fisher | EDTA | 1/200 |
| ST6GALNAC6 | HPA018890 | Sigma-Aldrich | EDTA | 1/10 |
